# Psychotherapeutic practice in paediatric oncology: four examples

**DOI:** 10.1054/bjoc.1999.0911

**Published:** 2000-01-18

**Authors:** D Oppenheim, O Hartmann

**Affiliations:** Departments of Paediatric Oncology, Institut Gustave Roussy, Villejuif, 94805, France; Psychiatry and Psycho-oncology Unit, Institut Gustave Roussy, Villejuif, 94805, France

**Keywords:** child, cancer, psychotherapy, non-compliance, parents, seque1

## Abstract

Psychotherapy, often used with children treated for a solid tumour, is seldom described. We present four examples of such therapies: a mother who refused enucleation for her 7-month-old boy; a boy's jealousy towards his sister who was being treated for a brain tumour; a teenager troubled by his scar; a 7-year-old boy embarrassed by the unconscious memory of his treatment when he was 5 months old. All names have been changed, for reasons of privacy. Psychotherapies aim to help children and parents to cope with the violent experience of having cancer, to recover their freedom of thought and decision-making concerning their life, their place in the family, their body image, their self-esteem, their identity. These descriptions of brief psychotherapy could help paediatricians to gain a more thorough understanding of the child's experience, to improve collaboration with psychotherapists and to confront clinical skills of psychotherapists. © 2000 Cancer Research Campaign


*British Journal of Cancer* (2000) **82**(2), 251–254
© 2000 Cancer Research Campaign
Article no. bjoc.1999.0911

Commentary

### Psychotherapeutic practice in paediatric oncology: four examples

**D Oppenheim[1,2] and O Hartmann[2]**

Departments of [1]Paediatric Oncology and [2]Psychiatry and Psycho-oncology Unit, Institut Gustave Roussy, 94805 Villejuif, France

**Summary** Psychotherapy, often used with children treated for a solid tumour, is seldom described. We present four examples of such therapies: a mother who refused enucleation for her 7-month-old boy; a boy's jealousy towards his sister who was being treated for a brain tumour; a teenager troubled by his scar; a 7-year-old boy embarrassed by the unconscious memory of his treatment when he was 5 months old. All names have been changed, for reasons of privacy. Psychotherapies aim to help children and parents to cope with the violent experience of having cancer, to recover their freedom of thought and decision-making concerning their life, their place in the family, their body image, their self-esteem, their identity. These descriptions of brief psychotherapy could help paediatricians to gain a more thorough understanding of the child's experience, to improve collaboration with psychotherapists and to confront clinical skills of psychotherapists. © 2000 Cancer Research Campaign

**Keywords:** child; cancer; psychotherapy; non-compliance; parents; sequel

A cancer is not only an object to be diagnosed and treated but also a capital rupture in a life history, a part of a body that lives and feels (Good, 1994), a set of words, a lived experience (Good, 1997) of a child who has conscious and unconscious thoughts. However, 'discussion of psychotherapy with the child with cancer is still a somewhat uncharted territory in the literature' (Sourkes, 1977). Many articles evaluate the efficiency of psychotherapy (Meyer and Mark, 1995; Kazak et al, 1996; Twillman and Manetto, 1998) but few describe them (Sourkes, 1982; Sourkes, 1995; Oppenheim, 1996, 1999). We present four examples of brief psychotherapeutic interventions taken from the 8000 interviews we had with over 1000 children treated for a solid tumour from 1988 to 1999.

### CASE 1: JEREMY'S MOTHER REFUSED TREATMENT (1 INTERVIEW)

The mother of a 7-month-old boy (Jeremy) with retinoblastoma, refused enucleation that the doctors considered mandatory. She had already been to several paediatric departments since the first symptoms and was aware that she had lost precious time. Behind her stubborn refusal I perceived major distress. She gave three reasons for her refusal:

1. she believed a miracle was going to occur
2. it was not true that Jeremy had a tumour
3. it was the doctor's fault.

I asked her whether she felt confronted with an impossible choice.

*Received 27 April 1999*
*Revised 9 September 1999*
*Accepted 14 September 1999*

*Correspondence to:* D Oppenheim

During the interview she revealed that her mother had been pregnant with twins, but this had not been discovered before the delivery. My patient was born first; her brother was born soon after but, in the general panic, they dropped him, and he died 2 days later. When she was 10 years old, her mother told her what had happened and added: 'it was just as well, because I wouldn't have been able to take care of two children'. My patient felt guilty about living; as if she had committed a sin and her mother had sacrificed her brother for her. She thought that Jeremy's illness was a punishment. She did not want to sacrifice one eye to save the other one, or even to save him. She was in revolt against her mother, just as she was against the doctors.

She preferred that Jeremy die then: it was better to lose him before strong relations set in. Then she said she preferred for a judge to take on the responsibility of deciding the enucleation; as a mother, she could not do so. I asserted that she nevertheless remained Jeremy's mother. A few days later she accepted surgery.

The result of her decision was that, over a year later, Jeremy was doing well and she was taking good care of him.

### Discussion

Aggressive treatments and parental depression have been associated with parental non-compliance. When the negative behaviour of parents cannot be understood or dissipated and when sensible advice and arguments prove inefficient, the reason may lie in the parents' own emotional and psychological state and in their own previous life experiences, including early childhood. Parents may cope with the child's present situation through the screen of the stressful or traumatic events they had lived and which are reactivated. The psychotherapist has to navigate between the present and past, which is very easily evoked by the parent. Problems linked to the past and the present must be solved together. Therapists should take into account the family history and the parent's difficulty in assuming their role, which can be linked to the relationship with their own parents when they were children. The search should be extended to reasons that may be both rational and irrational, conscious and unconscious. This can avoid severe conflicts (Brahams, 1993; Dyer, 1996).

### CASE 2: LOUISE'S JEALOUS BROTHER (1 INTERVIEW)

Louise, an 8-year-old girl, was being treated for a recurrent brain tumour. Her parents were anxious about the limited efficiency of the treatment, and were short-tempered with doctors because they thought the treatments were not strong enough. They were worried about Louise's brother, Peter, 5 years old, who showed signs of intense jealousy and had said: 'I want to die, so that you will take care of me'. I proposed an interview with him and his parents.

Peter's mother said that Louise's illness was something dirty. Then, Peter drew a machine, 'to clean dirty things off the streets, because the people in charge of the cleaning up did not do it well, so he was helping them'. (*His criticism of the doctors was similar to that of his parents, but he wanted to be active, to help his sister to get cured*.) Peter added that Louise was causing many problems in the family, which he hoped would end soon. (*He wanted her to disappear*.) His parents, horrified, accused him of being a monster. I explained his ambivalent thoughts: that jealousy is a common emotion between siblings, not contradictory to authentic love; that there is a difference between thoughts and deeds; and that if Peter went too far they could remind him of the reasonable limits of relations. Peter's parents felt they could assume their role of parents, and confirmed that they loved him as much as Louise and that it was difficult for all of them. I assured them all that Peter was a clever boy who was trying to understand the process of the illness and of the treatment, trying to help his sister and that it was important that he remain himself: neither a saint nor a monster. I reminded them that Louise was not a saint either.

### Discussion

'Siblings are a troubled group … given the potential for strong identification and severe rivalry' (Carpenter and Levant, 1994). In a situation where one child is ill, siblings have to strike a good balance between sacrificing themselves for the sick child (giving up friends, their interest in school, their expectations) or assuming the role of the egoistic one (the bad element, simply because they are not sick). They can feel guilty about their jealousy, remain angry with parents or doctors for not having avoided illness and may have very limited self-esteem. Caregivers must therefore give support to parents so that they can assume their role towards all their children: loving them and making them respect the usual rules (e.g. being sick does not mean that you can do as you wish and demand whatever you want).

Parents are not always aware of the intense and self-destructive jealousy of siblings, especially when its expression is discrete or playful. Caregivers should always ask parents about the siblings' situation (changes in behaviour and mood, lack of or excessive interest in school, somatic complaints, etc.) and give them advice. For instance, it is not always necessary that parents remain in the hospital at all times; they should try to attend to the everyday minor problems of siblings with the same amount of attention as that given to the medical state of the sick child. By asking about all children in a family, caregivers will acquire useful information and be able to usher parents into thinking about siblings.

A simple way to help siblings is to arrange a visit to the hospital and to introduce them to the doctors and nurses. A frightening and fantastic environment will be replaced by a more realistic image of the hospital; this measure may reassure healthy siblings that their place is preserved in the family.

The core of the psychotherapeutic care is in helping siblings express their ambivalent feelings towards the sick child, and in getting rid of feelings that can imprison them, especially when the sister or brother dies. Its objective is also to help parents maintain their parental role towards all their children.

### CASE 3: GEORGE'S (15-YEAR-OLD) DIFFICULTIES TO OVERCOME HIS EXPERIENCE OF LYMPHOMA (10 INTERVIEWS)

During his treatment, George thought he was going to die. We had our first discussion at that time. George was afflicted by an intense family history: his mother, a depressive alcoholic, had been treated for a cancer; he lived alone with her after his father left them. George's mother had tried to commit suicide on several occasions, and each time he had succeeded in saving her. When he found her dead he vowed he would no longer feel any emotion. He felt guilty for this as well as for the feeling that his birth had been too much for his mother.

During a subsequent interview, George admitted that he missed his mother and said that he had to live because he was the last one capable of wanting to and being able to live (he did not trust either his father or his ageing grandfather for that). His friends, whom he had met in the paediatric department, had since died. Despite this determination, he was attracted by death, and felt it would be difficult to live with Damocles' sword hanging over his head.

George wanted to become a pilot, like his father, and to perform aerial stunts. His father had once told him that if people knew their future they would jump out of a window and that his best memories were when he drove fast with his eyes closed. I advised George that he had ambivalent feelings towards his father: anger, admiration and love, and that he could accept to share common traits with him, but should try not to choose the dangerous ones. I said that people can be passive or active when confronted with risks. Risks can be calculated and some may be considered equivalent to a suicide. George wondered whether there was any point in being cured if there would be sequels.

At the end of his cancer treatment George was anxious and afraid of being alone. The hospital and the treatments had protected him – against illness as well as against life. He was also ambivalent towards the big scar, stretching across his chest. He could either exhibit it, like a trophy, or be ashamed and hide it. One week he dared to be stripped to the waist at the swimming pool, but found the experience exhausting. He felt he would never be able to show the scar when he was with a girl. He could ask a plastic surgeon to remove it, but he was ashamed of wanting to erase such an important part of his life, as he kept shame in relation to his mother. During his treatment, George behaved in a way he could be proud of. However, keeping the scar could signify his desire to prolong the illness, which was also the most intense and exciting part of his life, though he was happy to have recovered.

George was afraid that people would see only his scar, and nothing else. He did not want to be reduced to this scar, or to his experience of having had cancer. George's experience of cancer was a rich and complex existential experience, far more than surgery and chemotherapy. However, he did not feel capable of

*© 2000 Cancer Research Campaign*

explaining this to others: not only facts and rational explanations but all he had felt, thought and the meaning of this experience. Other people would never believe him, never understand (saying: 'you're kidding, it was not that terrible, you are alive'), would be fascinated, or would pity him. One day, he felt with relief a slight pain in his stomach: it could mean appendicitis. Thus, he would have a little, ordinary scar, and everybody would be lured to look at the little scar and forget the big one.

George's illness had been an ordeal for him, an initiation test to enter adulthood. If no relapse occurred, the family's doom would come to an end. This trial had given him something more than other teenagers or adults, and this was precious. He did not know what he could do with it, but this gain compensated for all he had lost through his illness. He did not even know what he had lost: his innocence, his adolescence? He had lost both of these before the illness, because of madness in his family, but the illness had given him a second chance: to lose them in his own process.

At last he discovered, with great relief, that all along either side of his scar there was a narrow, insensitive area. Other people might be trapped by the visible scar: that was left to them, to their curiosity, their fear. He was the only one who knew about this invisible, definitive strip: his private memory of cancer. He said we could stop our dialogue now. He now had the impression of being outside of the experience of cancer. When I met him several years later, George was well, had succeeded in his engineering studies and was living with a psychology student.

### Discussion

As for many adolescents cured of a cancer (Fritz and Williams, 1988; Smith et al, 1991), preoccupation concerning his physical condition, his body image, his identity, converged on this scar. It would have been an error to erase it with surgery, before understanding what the sequel meant to him. It was linked to George's past as well as to his present and to his future, to his illness as well as to his familial history, to his body as well as to the meaning and the value of his life. It was far more than a technical problem requiring a technical solution.

Patients may have two burdens to bear: a cancer and a distressful family history. This can induce excessive distress, or be a springboard towards challenging objectives in life. When their treatments are over, patients wonder whether they are really cured, but also what they are going to do with their life. Often, physical sequels have three meanings for them: impairment, Damocles' sword and a sign of a new and poorly accepted identity. Caregivers can help patients to extract themselves from the time and the world of illness and treatments if they can seriously consider what the illness, treatments and sequels signify for the patients and how this is related to the place these elements have taken in their lives.

### CASE 4: JOHN'S[1] MEMORY OF HIS EARLY CANCER (6 INTERVIEWS)

This 7-year-old child had been treated for a neuroblastoma when he was 5 months old. He was timid, obsessed with phobias, avoided contact with others, refused to go to school, remained close to his mother and complained of nightmares.

*First interview*: (I describe his free drawings and his comments. My comments are written in italics.) He drew a house with condemned windows (*the true description of his behaviour*).

*Second interview*: The windows were no longer condemned, but there was a 'STOP' sign in front of the garage (*inhibition*). I pointed out that the roof was not complete. He replied that the unprotected part belonged to the children, whereas the protected side belonged to Father Christmas. (*He believed that parents thought more about protecting themselves than about protecting him*.) Lightning (*illness*) had struck the house a long time ago and had left a hole in the roof which had been badly repaired. Sometimes the lightning would strike again making a further hole and everything had to be done all over again. The children were frightened and Father Christmas was oblivious to their needs. (*The effects of cancer were still present and it might reappear; his parents did not realize his fears; the treatment was not sufficient; the whole family had been affected by the upheaval provoked by cancer*.)

*Third interview*: A man, walking on his way home, was stopped by a barrier and a black hole crawling with rats: they had to be removed or else they would invade everything and even the apples would rot. He drew a long detour to get back home. (*The hole expresses the extreme limits of the psychological experience he had been through: the black hole of thought which cannot be rendered through images or expressed by words. The rats represent his effort to depict this experience but at the cost of fear and horror. The contamination of the fruit is disturbing: the body can be contaminated but also the children to be born*.)

*Fourth interview*: The apples had become eyes, a nose and a mouth, the elements of a smiling face. The road was clear but muddy. In a river, just below, sharks were killing each other (*who transmitted cancer to me and why?*). The man was saying that it was no longer his problem. (*He tries to lock the memory of cancer in the past*.) The man was picking the fruit with a pair of long tongs (*he had escaped from the danger but he continued to be wary*).

*Fifth interview*: A man was going to buy fish. (*Fishes had found their place in everyday life*.) A parasol was efficiently protecting the fishmonger from the sun. (*What a difference with the first roof!*) He drew grass which could not be walked on at that point because it had recently been planted but it was going to grow. (*He was optimistic but still prudent*.) He went inside a castle and found armour with weapons (*that protect but at great cost: a description of his symptoms*). He could not touch them because the weapons would fall off and seriously injure him. (*Treatments can be harmful to the child and not only to the cancer; the battle against cancer is not over*.) But they would soon be removed and there would be no danger anymore.

Inside the castle there were sketches on war, which had occurred a long time ago. (*The violent nature of the cancer has been transfigured by the psychological and artistic work. Violence is no longer on the road and an element of reality but placed in a frame. The images, endowed with the function of transmitting his subjective experience, had lost their direct link with cancer but without forgetting it, without circumscribing it in an enclosed area of the psyche. George expended the same effort to shift the drama from reality to the scene of the psyche, to make the experience of cancer lose its unbearable weight without making the place and the value it had in his life disappear*.)

*Sixth interview*: A barrier was preventing the man from pursuing his route: a crocodile had escaped from the zoo. A trap had been prepared; the crocodile would fall in it. Everything was going to go

[1]This extensive case has been published in Oppenheim D (1996) *L'enfant et le cancer, la traversée d'un exil*. Bayard: Paris. I thank the publisher for allowing me to quote excerpts from this case history.

© 2000 Cancer Research Campaign back to normal and the road would be clear again (*what a difference with the rats and the sharks*). The keepers (*his parents, his doctors*) knew what they had to do and they had the right tools.

*Second drawing*: The house was standing upright and firmly positioned. The tree on the left side (*the father's side*) had borne fruit. The tree on the right side (*the children's side*) was young and would bear fruits. (*The child had become the sanctuary of fertility; the cancer had not put an end to the family's destiny*.)

John and his father said that the interviews could come to an end. I met them both 1 year and 2 years later. John had no more problems and felt well and happy, and so did his parents.

### Discussion

Neither the passing of time nor medical cure may suffice to allow the children to emerge from the subjective experience they have endured (Canning et al, 1992). Consequently, even long after the end of treatment, paediatricians should be attentive to discrete psychic symptoms, such as loss of confidence, phobias, nightmares, excessive fantasies or a lack of fantasy and shyness, for they can reveal persistent entrapment in the patient's experience of cancer. This early experience left unconscious marks in John's psyche, revealed by symptoms, even when he had no recollection of it. Psychotherapeutic interviews revealed the upheaval in his body image, in his feelings regarding his identity, the image he had of the family structure and, most of all, how he was confronted with the limits of the conceivable, of that which cannot be rendered: the black hole of thought (Tustin, 1981).

John provides us with precious clues about what can help a child to cope with cancer. Children are not only in danger or harmed by cancer, they also have intense emotions, fears and fantasies. Caregivers can help parents to be attentive to these two aspects of the children's experience, so that they can continue to be valid interlocutors. Children express their fears and their lucid questions via fantasy. To avoid repressing these fears or being overwhelmed by them (which can block their ability to think), clowns (Oppenheim et al, 1997), art teachers and psychotherapists can help children express their experience precisely, but in a safe setting and in a context of fantasy. One of these fears is that both their body and their environment are contaminated by illness, in the present as well as in the future. Helping parents preserve their parental role will serve to dissipate these fears. Psychotherapeutic interviews helped John to retrieve his freedom of thought and pursue life at his own pace, along his own path.

### CONCLUSION

Psychotherapists help staff to gain a better knowledge of both childrens' needs and those of their relatives, and to improve their collective and their individual capacity to fulfil them. They help caregivers to detect early signs of psychosocial dysfunction, so that they can give advice and information and have pertinent discussions with children and their parents, or if need be, refer them to the psycho-oncologist. This article sheds light on four common situations in paediatric oncology, and more generally on the complex experiences that children go through. We are more acquainted with this experience through visible symptoms such as anxiety, depression, withdrawal, emotional and behavioural disturbances, than via its inner psychic process. Being aware of it will help caregivers to improve their diagnostic skills and their understanding of the children. Caregivers know that psychotherapies produce efficient results. If the psychotherapy process is understood, caregivers will be able to use the competence of psychotherapists more efficiently, at the right time, for the proper reasons, with the correct expectations. Psychotherapeutic interviews aim at helping children undergoing treatment or who have been treated for cancer, and their relatives, to overcome this difficult experience, to preserve or regain their place in the family, their body image, self esteem, identity and their ability to make choices concerning their life. This can be done in a short period of time.

### ACKNOWLEDGEMENTS

We thank L Saint Ange for editing, and an anonymous reviewer for pertinent advice.

### REFERENCES

Brahams D (1993) Religious objection versus parental duty. *Lancet* **342:** 1189–1190

Canning EH, Canning RD and Boyce WT (1992) Depressive symptoms and adaptative style in children with cancer. *J Am Acad Child Adolesc Psychiatry* **31:** 1120–1124

Carpenter PJ and LeVant CS (1994) Siblings adaptation to the family crisis of childhood cancer. In: *Pediatric Psycho-oncology*, Bearison DJ and Mulhern RK (eds), pp. 122–142. Oxford University Press: New York

Dyer C (1996) Mother wins right to refuse treatment for her child. *Br Med J* **313**: 1101

Fritz GK and Williams JR (1988) Issues of adolescent development for survivors of childhood cancer. *J Am Acad Child Adolesc Psychiatry* **27**: 712–715

Good BJ (1994) *Medicine, Rationality and Experience*. Cambridge University Press: New York, p. 337

Good BJ (1977) The heart of what's the matter. The semantics of illness in Iran. *Culture, Medicine and Psychiatry* **1**: 25–58

Kazak AE, Penati B, Boyer BA et al (1996) A randomized controlled prospective outcome study of a psychological and pharmacological intervention protocol for procedural distress in pediatric leukemia. *J Pediatr Psychol* **21**: 615–631

Meyer TJ and Mark MM (1995) Effects of psychosocial interventions with adult cancer patients: a meta-analysis of randomized experiments. *Health Psychol* **14**: 101–108

Oppenheim D (1996) *L'enfant et le cancer. La traversée d'un exil*. Bayard: Paris (in French).

Oppenheim D (1999) *Ne jette pas mes dessins à la poubelles. Dialogues avec Daniel, traité pour cancer, entre sa 6ème et sa 9ème année*. Seuil: Paris (in French)

Oppenheim D, Simonds C and Hartmann O (1997) Clowning on children's wards. *Lancet* **350**: 1838–1840

Smith K, Ostroff J, Tan O and Lesko L (1991) Alterations in self-perceptions among adolescent cancer survivors. *Cancer Invest* **9**: 581–587

Sourkes BM (1982) *The Deepening Shade: Psychological Aspects of Life Threatening Illness*. Pittsburgh University Press: Pittsburgh

Sourkes BM (1995) *Armfuls of Time*. Pittsburgh University Press: Pittsburgh

Sourkes BM (1998) Psychotherapy. In: *Psycho-oncology*, Holland JC (ed), pp. 946–953. Oxford University Press: New York

Tustin F (1981) *Autistic States in Children*. Routledge and Kegan: London

Twillman RK and Manetto C (1998) Concurrent psychotherapy and pharmacotherapy in the treatment of depression and anxiety in cancer patients. *Psycho-Oncology* **7**: 285–290

 *© 2000 Cancer Research Campaign*

